# Management of a Complicated Ruptured Infected Pseudoaneurysm of the Femoral Artery in a Drug Addict

**DOI:** 10.1155/2012/434768

**Published:** 2012-11-28

**Authors:** Emmanouil Psathas, Stella Lioudaki, Fotios-Filippos Karantonis, Petros Charalampoudis, Othon Papadopoulos, Chris Klonaris

**Affiliations:** ^1^Second Department of Propaedeutic Surgery, Laiko General Hospital of Athens, Athens University Medical School, 17 Ag. Thoma Street, 11427 Athens, Greece; ^2^Department of Plastic Surgery, Andreas Syngros Hospital, 5 Dragoumi Street, 16121 Athens, Greece; ^3^Vascular Division, Second Department of Propedeutic Surgery, Laiko General Hospital of Athens, Athens University Medical School, 17 Ag. Thoma Street, 11527 Athens, Greece

## Abstract

Infected pseudoaneurysm of the femoral artery represents a devastating complication of intravenous drug abuse, especially in the event of rupture. Operative strategy depends upon the extent of arterial injury and the coexistence of infection or sepsis. Options range from simple common femoral artery (CFA) ligation to complex arterial reconstruction with autologous grafts (arterial, venous, or homografts). We report herein the management of a 29-year-old male patient who was urgently admitted with a ruptured pseudoaneurysm of the right CFA, extending well above the inguinal ligament. Multidisciplinary approach with multiple arterial reconstructions and subsequent coverage of the tissue defect with a rectus abdominis musculocutaneous flap transposition was performed.

## 1. Introduction

Intravenous drug abuse represents a growing worldwide social and medical problem. Various vascular complications, such as deep venous thrombosis (DVT), infectious pseudoaneurysms, venous gangrene, and arterial embolization, can occur in drug addicts, frequently requiring prompt management [1]. The incidence of infected pseudoaneurysms of the femoral or external iliac artery in this subgroup of patients is quite high and various procedures for limb salvage have been described [2]. We herein present a case of a young heroin drug user, who presented to our department with signs of sepsis, bleeding, and a pulsatile mass in the right lower quadrant of the abdomen.

## 2. Case Report

A 29-year-old male patient was admitted to the emergency department with a bleeding pulsatile mass of the right groin and pain in the right lower abdominal quadrant and hypogastrium. Upon admission, the patient was confused, disorientated, and febrile. Past history included hepatitis C and heroin abuse over the last 10 years. Vital signs included a blood pressure of 130/70 mmHg, a heart rate of 110 pulses per minute and a body temperature of 38.7 Celsius degrees. Clinical examination revealed a painful, pulsatile mass extending above the right inguinal ligament to the midline. There were extensive skin and soft tissue necrosis and a fistulous track draining blood and pus. Laboratory investigation showed anemia (Ht = 34%), marked leukocytosis (24.65 K/*μ*L), and an elevated CRP (186 mg/dL). After insertion of a central venous line catheter for fluid resuscitation, a diagnostic duplex ultrasound was performed, which revealed a large pseudoaneurysm of the right common femoral artery (CFA), extending above the inguinal ligament and surrounded by a large extraperitoneal abscess. Abdominal and thigh computed tomography scan demonstrated a 20-millimeter pseudoaneurysm of the right external iliac and common femoral artery, with marked inflammatory fluid collection.

The patient was transferred to the surgical ward where intravenous administration of antibiotics was initiated. Digital subtraction angiography (DSA) of the abdominal aorta, iliac, and peripheral arteries was performed via a left brachial approach, in an attempt to primarily exclude the pseudoaneurysm with a stent graft before surgical debridement and drainage of the abscess. Due to profound kinking and compression of the external iliac artery by the surrounding abscess, an attempt to cross the lesion antegrade proved fruitless. A second attempt to exclude the pseudoaneurysm retrograde via femoral cutdown also failed, despite successful crossing of the lesion with a 0.035′′ J-wire because the stent graft could not be advanced beyond the external iliac artery. 

**Figure 1:**
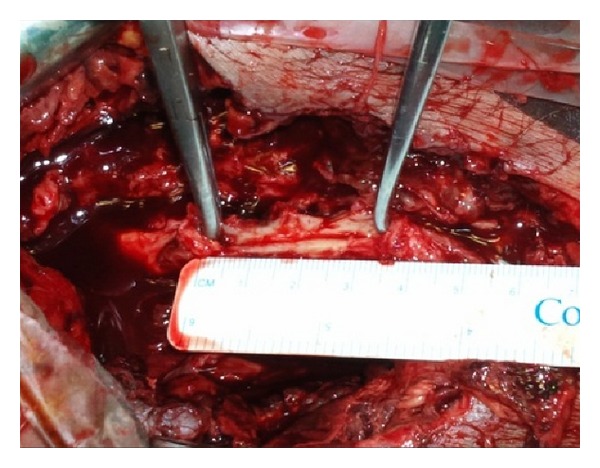
After proximal and distal control, careful dissection of the common femoral and external iliac artery revealed a 3 cm longitudinal arterial defect.

**Figure 2:**
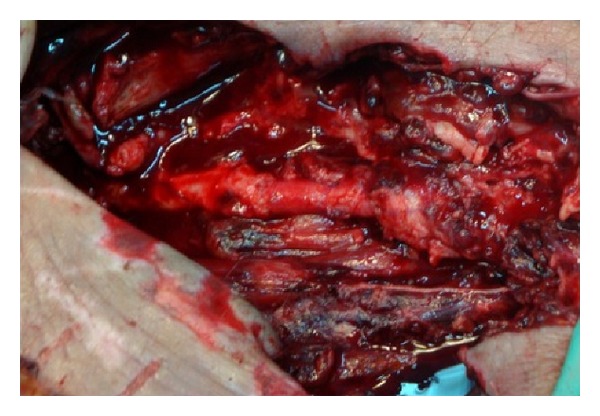
Internal iliac interposition arterial graft with end-to-end anastomosis.

We next opted for an open approach. A midline abdominal incision was carried out and the aortic bifurcation and the right external and internal iliac artery were dissected and controlled with elastic tapes. The CFA was exposed via a longitudinal incision in the groin. After heparin administration and cross-clamping of the external iliac and the CFA, the right extraperitoneal cavity was explored and a large amount of pus was evacuated. Careful dissection of the distal external iliac artery towards the CFA revealed a 3 cm long defect in the anterior wall of the artery (Figure 1). Due to the unavailability of any autologous veins, the right internal iliac artery was harvested from its origin to its first pelvic branch and used as an interposition graft from the external iliac to the CFA in an end-to-end fashion. Based upon our previous experience with the use of the internal iliac artery [3], we considered this to be the best option for this patient (Figure 2). Cultures obtained from the abscess were positive for *Streptococcus* and *Klebsiella*. Due to extensive necrosis of the overlying skin and soft tissues, the femoral wound was partially closed.

**Figure 3:**
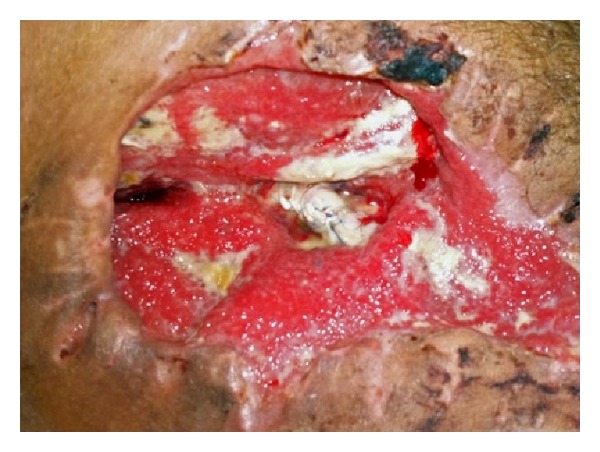
PTFE patch wrapped around the arterial graft to secure the anastomosis. Notice the granulated tissue in secondary healing.

On the 10th postoperative day, the patient suffered bleeding from a leak in the graft anastomosis and was emergently taken to the operating room, where a PTFE patch was wrapped over the arterial graft to secure the anastomotic site (Figure 3). Unfortunately, ten days later, the patient suffered a second, massive bleeding from the right femoral artery, due to patch rupture, and was reoperated urgently due to imminent hemorrhagic shock. Immediate clamping of the femoral artery was performed to stop the bleeding, and, after resuscitation, the right femoral artery was ligated. An extra-anatomical right subclavian-popliteal bypass was then carried out using an 8 mm PTFE graft. Postoperatively, the patient maintained a good peripheral arterial flow and, after daily wound care and surgical debridement, reconstructive surgery was planned to cover the tissue defect of the right groin.

**Figure 4:**
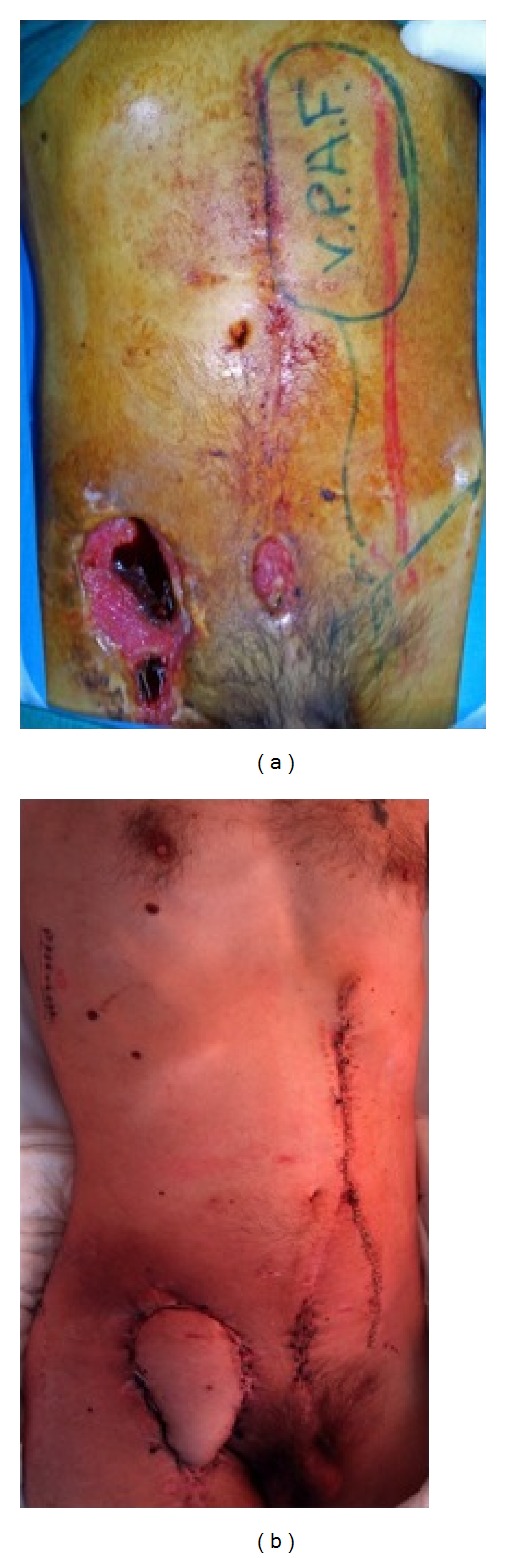
Operative planning (a) and final result (b) after the use of left rectus abdominus muscle as a transposition graft to the right groin.

A contralateral, distally based rectus abdominis musculocutaneous flap was harvested in order to cover the affected right groin area. Due to previous manipulations of the iliac and femoral vessels we opted for the rectus abdominis muscle instead of the tensor fascia lata (TFL) flap, routinely used otherwise. The distal third of the flap was harvested as a myocutaneous flap in order to reconstruct the right groin area, whereas the proximal two thirds consisted solely of the rectus abdominis muscle separated from its fascia. After ligating the superior epigastric vessels and dividing the proximal insertion of the muscle, the flap was meticulously raised from the posterior fascia and passed through a suprapubic tunnel in order to cover the right groin defect. Subsequently, the myocutaneous portion of the flap was fixed in place. A mesh was used to cover and strengthen the lower part of the abdomen in order to minimize herniation (Figure 4). The patient was mobilized on the 5th postoperative day, while the rest of his postoperative course was uneventful. He was discharged after 64 days of hospitalization on double antiplatelet therapy (aspirin 100 mg plus clopidogrel 75 mg daily) and satisfactory wound healing. The extra-anatomic graft remains patent one year postoperatively.

## 3. Discussion

Limb salvage procedures in patients with ruptured infected pseudoaneurysms of the groin are challenging and often require special technical considerations. Simple ligation of the external iliac or CFA without revascularization represents the most common treatment approach. Such a management however carries considerable risk of severe claudication, critical ischemia, and subsequent amputations [4] and therefore should be preserved for cases where revascularization cannot be applied.

Endovascular pseudoaneurysm exclusion with primary stent-grafting and secondary surgical debridement have been previously described as a less invasive hybrid approach, providing bleeding control and maintaining a distal perfusion of the affected limb [5–8]. Such a hybrid technique albeit carries some risks related to the placement of a stent graft in an already infected area and should be used only as a bridge to open surgery [7]. In our case such an approach proved unsuccessful, due to failure to advance the stent graft either antegrade or retrograde, respectively. 

In situ surgical arterial reconstruction represents an alternative option in order to repair the arterial deficit and maintain perfusion to the affected limb. Synthetic grafts should be avoided due to the increased risk of infection and recurrent bleeding [9], while autologous veins may be unavailable in this subgroup of patients because of chronic drug abuse and possible recurrent episodes of DVT [3]. Based on our previous experience [3], the internal iliac artery can be used as an autologous conduit as it carries several advantages over vein grafts. In this particular patient however, initial in situ arterial reconstruction with the use of the ipsilateral internal iliac artery as a conduit proved ineffective since the severe infection setting led to suture line disruption and recurrent bleeding. Of note, this is the first case of such a complication in the authors' experience after 21 consecutive patients treated successfully with the internal iliac artery as autologous conduit.

Ligation of the defected CFA and limb reperfusion via an extra-anatomical bypass graft is an alternative option. The transobturator foramen route is used preferentially for iliofemoral bypass grafts with good results; however such an approach was not possible in our case due to regional conditions. Thus, an extra-anatomic subclavian-popliteal bypass was performed. Such a long bypass graft is not optimal from a hemodynamic point of view but it can be effective in salvaging the limb of such patients [10], who are most commonly young individuals with poor previously developed collateral circulation and therefore are not good candidates for simple CFA ligation without revascularization.

## 4. Conclusion

This case stresses the challenges a vascular surgeon may face during arterial reconstruction for infected femoral pseudoaneurysm repair. Simple CFA ligation bears a significant risk for severe claudication or limb loss, especially in young cohorts, thus delineating the need for adjunct revascularization procedures. When in situ arterial reconstruction with homologous grafts cannot be applied, extra-anatomic bypass graft is a conventional, but still effective treatment and should be considered as a useful option. Reconstructive surgery in order to cover tissue loss resulting from extensive skin and soft tissue necrosis is necessary to achieve full recovery and minimize morbidity in this subset of patients.
